# Dronedarone synergizes with colistin against planktonic and biofilm forms of multidrug-resistant Gram-negative pathogens

**DOI:** 10.1128/spectrum.03006-25

**Published:** 2025-12-23

**Authors:** Mohamed F. Mohamed, Somaia M. Abdelmegeed, Abdallah S. Abdelsattar, Ahmed A. Abouelkhair, Nader S. Abutaleb, Mohamed N. Seleem

**Affiliations:** 1 Department of Biomedical Sciences and Pathobiology, Virginia-Maryland College of Veterinary Medicine, Virginia Tech 1757, Blacksburg, Virginia, USA; 2 Center for One Health Research, Virginia Tech 1757, Blacksburg, Virginia, USA; Purdue University, West Lafayette, Indiana, USA

**Keywords:** Gram negative, colistin, dronedarone,

## Abstract

Infections caused by multidrug-resistant Gram-negative pathogens (MDR-GNP) are associated with high mortality, prolonged hospital stays, and increased healthcare costs. The treatment of these infections is complicated by the scarcity of new antibiotics and rising resistance to existing drugs. Colistin is often considered a last-resort therapy for MDR-GNP, but its clinical use is limited by significant nephrotoxicity and neurotoxicity. These challenges highlight the need for alternative strategies to enhance the effectiveness of current antibiotics. One promising approach is the use of combination therapy, which can potentiate antimicrobial activity while mitigating toxicity. We screened ~3,400 FDA-approved compounds and identified dronedarone, an antiarrhythmic with a well-established safety profile, as a potent enhancer of colistin activity against several MDR-GNP, including *Pseudomonas aeruginosa*, *Acinetobacter baumannii*, *Klebsiella pneumoniae*, and *Escherichia coli*. The colistin/dronedarone (COL/DRO) combination restored colistin efficacy *in vitro* and significantly reduced bacterial burden in a *Caenorhabditis elegans in vivo* model. Mechanistic studies revealed that colistin disrupts the outer membrane, facilitating dronedarone entry, which then permeabilizes and depolarizes the inner membrane, leading to cell death. This synergistic mechanism likely explains the potent activity observed. Additionally, the COL/DRO combination completely eradicated preformed biofilms of all tested pathogens. These findings suggest that dronedarone may be effectively repurposed to enhance colistin therapy, offering a promising strategy to combat life-threatening infections caused by MDR-GNP.

**IMPORTANCE:**

Antibiotic resistance in dangerous Gram-negative bacteria is a growing global health crisis, leaving doctors with very few treatment options. Colistin is often the last available antibiotic for these infections, but its effectiveness is limited by serious side effects including nephrotoxicity and neurotoxicity. Our study shows that dronedarone, a heart medication already approved for human use, can make colistin much more effective against highly resistant bacteria. By working together, these two drugs kill bacteria that neither drug can eliminate alone, including those that form hard-to-treat biofilms. The combination also proved effective in an *in vivo* infection model, showing promise beyond the laboratory. Because dronedarone has a known safety record in people, this approach could be developed more quickly than entirely new antibiotics. These findings highlight a practical strategy to repurpose existing medicines to strengthen current antibiotics and fight life-threatening, drug-resistant infections.

## INTRODUCTION

The increasing prevalence of multidrug-resistant Gram-negative pathogens (MDR-GNP) presents a significant and daunting challenge for healthcare providers (1, 2). Infections caused by MDR-GNP are associated with elevated mortality rates, prolonged hospital stays, and increased healthcare costs (3, 4). The challenge of treating such infections is exacerbated by the lack of new antibacterial drugs and the growing resistance to existing treatments (2, 3). Colistin (COL) is now often viewed as the last resort treatment option for MDR-GNP infections (5). While COL is highly effective, its clinical use is hampered by significant downsides and serious dose-limiting adverse effects, including nephrotoxicity and neurotoxicity (5, 6). Moreover, the emergence of COL resistance in these pathogens, either through spontaneous genetic mutation or the spread of mobile antibiotic resistance genes on plasmids, presents an urgent threat to modern medical practice (7).

**Fig 1:**
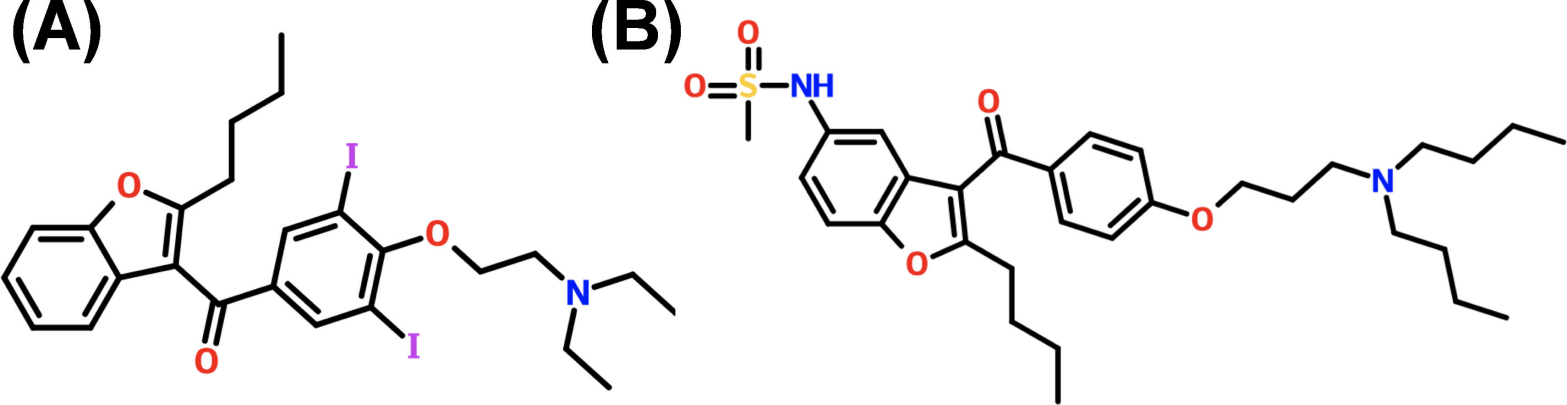
Structure of amiodarone (**A**) and dronedarone (**B**).

The primary adverse effect of COL is acute kidney injury, which can occur in up to 70% of patients and is linked to higher mortality rates in hospital and intensive care settings (8–11). The cumulative dose of COL correlates with the degree of kidney damage, suggesting that reducing the length of treatment may help lower the risk of nephrotoxicity (6, 12). In response to these challenges, a strategic approach involves combination therapy, utilizing a potentiator drug to enhance the effectiveness of COL. This approach aims to lower the required dose of COL, thereby reducing toxicity and improving efficacy. The proposed work emerged from discoveries of the potent COL re-sensitization activities and the numerous advantageous qualities of dronedarone against highly MDR-GNP. Dronedarone (DRO), an FDA-approved antiarrhythmic drug, is prescribed 800 mg/daily for managing atrial flutter and atrial fibrillation (13). DRO is a modified analog of amiodarone, with structural differences that include the removal of iodine and addition of a methane-sulfonyl group (Fig. 1). These modifications have improved the safety profile of DRO in comparison to amiodarone (13, 14). There is growing interest in repurposing antiarrhythmic drugs for infectious diseases, as amiodarone is currently used in clinical settings to treat chronic Chagas disease and disseminated cutaneous leishmaniasis (15–17). Notably, similar therapeutic effects have been observed with DRO (17, 18). In addition to its efficacy, DRO shows broad distribution across key organs such as the lungs, kidneys, heart muscle, and liver (14), further supporting its potential as a treatment option for infectious diseases. Our preliminary data demonstrated that DRO works synergistically through a novel mechanism with COL against carbapenem-resistant MDR-GNP, including *Pseudomonas aeruginosa*, *Acinetobacter baumannii*, *Klebsiella pneumoniae*, and *Escherichia coli*. Additionally, DRO’s unique mechanism of targeting bacterial membranes offers several advantages: it reduces the likelihood of resistance development and completely eradicates established biofilms of all tested MDR-GNP. This highlights the potential of the COL/DRO combination as a future therapeutic option for persistent bacterial infections. This research focuses on evaluating DRO as a potentiator to enhance COL’s effectiveness, thereby decreasing the required dosage, reducing toxicity, and improving efficacy against both planktonic and biofilm forms of MDR-GNP.

## MATERIALS AND METHODS

### Library screening and identification of DRO

To identify a drug capable of potentiating the antibacterial activity of COL, we screened ~ 3,400 FDA-approved drugs and clinical compounds (MCE HY-L066) against *P. aeruginosa* PAO1 in the presence or absence of COL 1/4 × MIC. The assay followed Clinical and Laboratory Standards Institute (CLSI) guidelines (19–22) with minor modifications. Briefly, *P. aeruginosa* PAO1 cultures were cultured on TSA and incubated at 37°C for 24 h. Colonies were suspended in CA-MHB and adjusted to a final inoculum of approximately 5 × 10⁵ CFU/mL. The bacterial suspension was dispensed into 96-well microtiter plates containing test compounds at a final concentration of 4 µM in the presence or absence of COL 1/4 × MIC. Plates were incubated at 37°C for 24 h. Dimethyl sulfoxide (DMSO) served as the negative control, and amikacin was included as reference standards. Bacterial growth inhibition was assessed using an x spectrophotometer at OD_600_ spectrophotometrically, and minimum inhibitory concentrations (MICs) were determined via broth microdilution. MIC was defined as the lowest concentration with complete inhibition of visible growth (23).

### Antibacterial assay

The minimum inhibitory concentrations (MIC) of the tested compounds were evaluated using the broth microdilution method, adhering to the Clinical and Laboratory Standards Institute (CLSI) guidelines (19, 20). The MIC experiments utilized a starting bacterial inoculum of 5 × 10⁵ colony-forming units (CFU) per milliliter in Mueller Hinton broth (MHB). The drugs were added into polystyrene 96-well plates at specified concentrations. The MIC was identified as the lowest concentration at which bacterial growth was visibly suppressed (23).

### Checkerboard assay

The synergistic activity of DRO in combination with COL was assessed following previously established protocols (21, 22, 24–27). Briefly, bacterial suspensions were prepared for *P. aeruginosa* NR-51547, *A. baumannii* ATCC BAA-1605, *E. coli* ATCC BAA-2452, and *K. pneumoniae* ATCC BAA-1705 (Table 1) to match the 0.5 McFarland standard. These suspensions were further diluted in MHB to obtain a final bacterial inoculum of 5 × 10⁵ CFU/mL. COL and DRO were added to 96-well plates at varying concentrations, and the plates were incubated for 18 h at 37°C. Bacterial growth was subsequently quantified by measuring optical density at 600 nm using a microplate reader (Synergy H1, BioTek, USA). The percentage of bacterial growth inhibition was analyzed and visualized as a heat map. To determine interaction outcomes, the fractional inhibitory concentration index (FICI) is calculated using the formula: FICI = (FIC A) + (FIC B) = (MIC AB)/(MIC A) + (MIC BA)/(MIC B). In this equation, (MIC AB) is MIC of drug A in combination, and (MIC A) is the MIC of drug A alone. Similarly, (MIC BA) is the MIC of drug B in combination, and (MIC B) is the MIC of drug B alone. Because dronedarone alone showed no measurable antibacterial activity at concentrations up to 256 µg/mL, a concentration of 512 µg/mL was assigned for the FICI calculation. FICI values were interpreted as follows: FICI ≤ 0.5 indicated a synergistic effect. FICI > 0.5 and ≤ 1.25 represented an additive effect. FICI > 1.25 and ≤ 4 suggested indifferences. FICI > 4 signified an antagonistic interaction. The checkerboard assay was performed independently on two separate occasions to ensure result reliability.

**TABLE 1:** Isolate description of Gram-negative pathogens used in the study

Isolate	Isolate description
*P. aeruginosa* PAO1	Common laboratory and whole-genome sequenced isolate
*P. aeruginosa* NR51547	Isolated in 2004 from a human urine sample in the United States. Resistant to aztreonam, ciprofloxacin, cefepime, gentamicin, imipenem, levofloxacin, meropenem, tobramycin, and piperacillin/tazobactam
*A. baumanii* ATCC BAA-1605	Isolated from sputum of military personnel returning from Afghanistan entering a Canadian hospital 2006. A multidrug-resistant strain resistant to ceftazidime, gentamicin, ticarcillin, piperacillin, aztreonam, cefepime, ciprofloxacin, imipenem, and meropenem
*E. coli* ATCC BAA-2452	A whole-genome sequenced drug-resistant bacterium that was isolated in Pakistan. This strain is resistant to ertapenem and imipenem and was confirmed by PCR to contain the blaNDM gene
*K. pneumoniae* ATCC BAA-1705	Isolated from urine. This strain is a *Klebsiella pneumoniae* carbapenemase (KPC) producer
*P. aeruginosa* 1125	Isolated from patient in Leeds, UK, 1997. Colistin resistance isolate isolated from cystic fibrosis patients (28)
*P. aeruginosa* 1131	Isolated from patient in Leeds, UK, 1999. Colistin resistance isolate isolated from cystic fibrosis patients (28)
*P. aeruginosa* 1133	Isolated from patient in Leeds, UK, 2000. Colistin resistance isolate isolated from cystic fibrosis patients (28)
*P. aeruginosa* 1571	Isolated from patient in Copenhagen, Denmark, 2002/1995. Colistin resistance isolate isolated from cystic fibrosis patients (28)
*E. coli* ΔbamBΔtolC	With a ΔbamBΔtolC deletion (29), resulting in high permeability to small molecules due to the absence of BamB (a crucial element of the β-barrel assembly mechanism for OM proteins) and TolC (an efflux channel in the outer membrane),
WT *E. coli*	Wild-type *E. coli* (29)

### Time kill assay

The time-kill assay was performed following established methods (30, 31). Cultures of *P. aeruginosa* NR-51547, *A. baumannii* ATCC BAA-1605, *E. coli* ATCC 2452, and *K. pneumoniae* ATCC BAA-1705 were grown overnight in MHB. Bacteria were subsequently diluted in fresh MHB and incubated under aerobic conditions at 37°C until the cells reached the logarithmic growth phase (determined at OD₆₀₀ = 0.2). The bacterial cultures were further diluted to achieve a final inoculum of 5 × 10⁵ CFU/mL in MHB. Treatments were added to bacterial cultures as follows: COL at 0.25× MIC, DRO at a concentration of 8 µg/mL, and their combination. Additionally, COL at 2× MIC was tested as a standalone treatment. The bacterial cultures were incubated aerobically at 37°C. At predetermined time intervals, aliquots were extracted, serially diluted using phosphate-buffered saline (PBS), and plated in triplicate onto tryptic soy agar (TSA) plates. The plates were incubated for 24 h at 37 °C, after which colony-forming units (CFUs) were enumerated to determine bacterial survival over time.

### Cytotoxicity assay

The cytotoxic effects of the compounds, both individually and in combination, on Vero cells (monkey kidney epithelial cells) were assessed following established protocols (27, 30). In summary, Vero cells were seeded into 96-well plates using Dulbecco’s modified Eagle medium (DMEM) supplemented with 10% fetal bovine serum (FBS). The cells were allowed to grow for 24 h at 37°C in a humidified incubator with 5% CO₂. After 24 h, the existing medium was replaced with fresh DMEM containing serial dilutions of the test compounds. The solvent alone was used as a negative control. The cultures were incubated for an additional 24 h under the same conditions. To evaluate cell viability, the MTS reagent was added to the wells 4 h prior to reading the absorbance at 490 nm using a microplate reader (Synergy H1, BioTek, USA). All experiments were performed in triplicate to ensure reproducibility and accuracy of the results.

### Hemolysis assay

The hemolytic activity of the compounds was assessed following established protocols (26, 30, 32). Fresh human red blood cells (RBCs) were purchased from Innovative Research Inc (MI, USA) and subjected to centrifugation at 2,000 rpm for 5 min to form a pellet. The RBC pellet was washed three times with PBS. An 8% (vol/vol) RBC suspension was prepared in PBS, and 50 µL of this suspension was transferred into each well of a 96-well plate. To this, 50 µL of the test compounds at varying concentrations, prepared in PBS, was added to achieve a final RBC concentration of 4% (vol/vol) in each well. PBS alone was used as the negative control, while 0.1% Triton X-100 served as the positive control for complete hemolysis. The plate was incubated at 37°C for 1 h to allow for any potential hemolytic activity. Following incubation, the plate was centrifuged at 1,000 rpm for 5 min at 4°C to separate the intact RBCs from the supernatant. Carefully, 75 µL of the resulting supernatants was transferred to a new sterile 96-well plate. Hemolysis was quantified by measuring the absorbance at 405 nm using a microplate reader (Synergy H1, BioTek, USA). The percentage of hemolysis was calculated relative to the 100% hemolysis control (0.1% Triton X-100). All experiments were performed in triplicate to ensure consistency and reproducibility.

### Outer membrane permeabilization assay

The ability of compounds to disrupt bacterial outer membrane integrity was evaluated using the hydrophobic fluorescent probe 1-N-phenylnaphthylamine (NPN) (30, 31). Cultures of *E. coli* and *P. aeruginosa* were grown to the logarithmic phase, harvested by centrifugation at 4,000 × *g* for 10 min, and washed to remove residual media. The bacterial suspension was then adjusted to an optical density (OD₆₀₀) of 0.5. A 100 µL aliquot of the prepared bacterial suspension was transferred into black-walled 96-well plates. The fluorescent dye NPN was added to the bacterial suspension to achieve a final concentration of 10 μM. Test compounds were introduced at predetermined concentrations, and each condition was performed in triplicate to ensure accuracy. Fluorescence measurements were recorded using a microplate reader (Synergy H1, BioTek, USA), with the excitation wavelength set at 350 nm and the emission wavelength at 420 nm. The degree of fluorescence indicated the extent of outer membrane permeabilization, as NPN fluoresces more strongly upon entry into the compromised outer membrane. All experiments were performed in three independent biological replicates to confirm reproducibility and reliability of the results.

### Inner (cytoplasmic) membrane permeabilization and depolarization assay

The impact of compounds on the integrity and polarization of the bacterial inner (cytoplasmic) membrane was assessed using two fluorescent dyes: propidium iodide (PI) for membrane permeabilization and 3,3′-dipropylthiadicarbocyanine iodide (DiSC3(5)) for membrane depolarization. This procedure followed established methods described in the literature (30, 31). Cultures of *E. coli* ΔbamBΔtolC were grown to the logarithmic phase, harvested by centrifugation at 4,000 × *g* for 10 min, and washed to remove any residual medium. The bacterial suspension was standardized to an optical density (OD₆₀₀) of 0.2. A 100 µL aliquot of the prepared bacterial suspension was transferred into black-walled 96-well plates. Fluorescent dyes were added to the wells at a final concentration of 10 µM each. The test compounds were introduced at specified concentrations, and each condition was carried out in triplicate for accuracy. Bacteria treated with DMSO, the compound solvent, served as the negative control. Fluorescence was measured using a microplate reader (Synergy H1, BioTek, USA). For propidium iodide, excitation and emission wavelengths were set at 585 nm and 620 nm, respectively, while DiSC3(5) fluorescence was monitored at excitation and emission wavelengths of 622 nm and 670 nm. All experiments were performed in three independent biological replicates to ensure reproducibility and reliability of the results.

### Evaluation of COL/DRO combinations on bacterial biofilms

The effectiveness of COL/DRO combinations in disrupting bacterial biofilms was assessed using a microtiter plate biofilm formation assay based on established protocols (30, 31). Cultures of *P. aeruginosa* NR-51547, *A. baumannii* ATCC BAA-1605, *E. coli* ATCC 2452, and *K. pneumoniae* ATCC BAA-1705 were grown overnight in MHB. Overnight cultures were diluted 1:100 in biofilm growth medium, which consisted of M63 minimal medium supplemented with magnesium sulfate, glucose, and casamino acids to support biofilm development. Biofilms were allowed to develop for 24 h. After the initial incubation period, wells were gently washed twice with PBS to remove any non-adherent planktonic cells. Fresh medium containing the test compounds— COL and DRO at specified concentrations—was then added, and the plates were incubated for an additional 24 h to assess treatment effects. Following this second incubation, the wells were washed three times with distilled water to eliminate residual medium and unattached cells. The biofilms were air-dried and stained using a 0.1% crystal violet solution for 30 min to visualize the adherent biomass. Excess crystal violet was removed by washing with distilled water, and the remaining dye bound to biofilms was solubilized using 30% glacial acetic acid. The extent of biofilm formation and disruption was quantified by measuring absorbance at 550 nm using a microplate reader (Synergy H1, BioTek, USA). To ensure accuracy and reproducibility, each experiment was performed in three biological replicates and repeated independently twice.

### *In vivo* efficacy of COL/DRO combinations in a *Caenorhabditis elegans* infection model

The temperature-sensitive sterile mutant strain *C. elegans* AU37 [sek-1(km4); glp-4(bn2) I] was used to test the efficacy of drugs *in vivo* as previously described (33, 34). Briefly, adult worms were grown for 5 days at 15°C (to permit adult worms to lay eggs) on NGM agar plates seeded with a lawn of *E. coli* OP50. The eggs were harvested by bleaching and maintained for 24 h at room temperature with gentle agitation for hatching. Hatched larvae were transferred to a new NGM plate seeded with *E. coli* OP50 and were kept at room temperature until worms reached adult stage of growth. Adult worms were collected and washed three times with PBS, to remove *E. coli*, before infection with carbapenem-resistant *P. aeruginosa* NR 51547. After infection, worms were collected and washed with PBS three times. Worms (~30 per sample) were treated with DRO (12 µg/mL), COL (1 µg/mL), or their combination. To assess the bacterial load in worms, worms were washed three times with PBS and subsequently lysed by the addition of 200 mg of 1.0-mm silicon carbide particles (Biospec Products, Bartlesville, OK) to each tube and vortexing for 5 min. Bacteria were plated onto *Pseudomonas* isolation agar. Plates were incubated for 16 h at 37°C, and bacterial colonies were counted.

### Statistical analyses

All statistical analyses were conducted using GraphPad Prism 10 software (GraphPad Software, La Jolla, CA, USA). For comparisons involving two groups, a two-tailed unpaired Student’s *t*-test was used to determine statistical significance. When comparing three or more groups, a one-way analysis of variance (ANOVA) was performed, followed by Tukey’s post hoc test for multiple comparisons. Results were considered statistically significant if the *P*-value was less than 0.05.

## RESULTS

### Library screening and identification of DRO

To identify compounds that could enhance the antibacterial activity of COL, we screened approximately 3,400 FDA-approved drugs and clinical molecules against *P. aeruginosa* PAO1. Among the screened compounds, DRO emerged as a potent enhancer of COL activity with a fractional inhibitory concentration index (ΣFICI) value of 0.069, indicating strong synergy. In the presence of 0.031 μg/mL COL, the MIC of DRO decreased to 4 μg/mL, translating to at least a 16-fold reduction in the concentration of COL required to inhibit bacterial growth. In contrast, DRO alone displayed no intrinsic antibacterial activity, with an MIC greater than 256 μg/mL. These results demonstrate that while DRO is inactive on its own, it markedly potentiates COL activity, suggesting that it may act through a mechanism that facilitates or enhances COL-mediated bacterial killing. This initial screening result prompted further evaluation of DRO’s synergistic behavior across clinically relevant multidrug-resistant Gram-negative pathogens (MDR-GNP).

### DRO works synergistically with COL against carbapenem-resistant MDR-GNP

**Fig 2:**
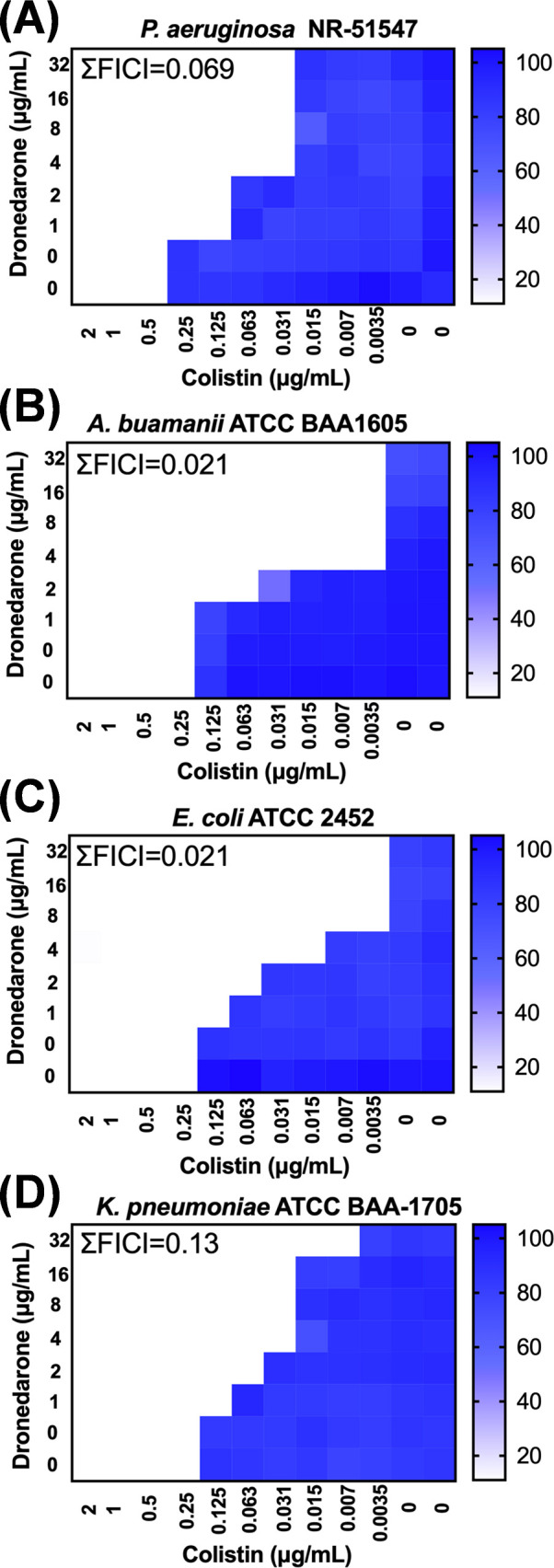
Heat map illustrating the synergistic effect of dronedarone (DRO) in combination with colistin (COL) against four clinically significant carbapenem-resistant MDR-GNP. (**A**) *P. aeruginosa* NR51547, (**B**) *A. baumannii* BAA-1605, (**C**) *E. coli* BAA-2452 and (**D**) *K. pneumoniae* BAA-1705. White color indicates no growth. Experiments were repeated twice, and the mean was presented.

Following the initial identification, we tested the interaction between COL and DRO against four major carbapenem-resistant MDR-GNP: *P. aeruginosa* NR51547, *A. baumannii* BAA-1605, *K. pneumoniae* BAA-1705, and *E. coli* BAA-2452. Checkerboard assays revealed that DRO alone (MIC > 256 µg/mL) had no effect on any tested isolate, but in combination with COL, potent synergy was observed with ΣFICI values ranging from 0.021 to 0.13 (Fig. 2). These low ΣFICI values indicate robust synergistic interactions across all tested species. Importantly, the MIC of DRO decreased to 4 μg/mL in the presence of 0.031–0.003 μg/mL COL, corresponding to up to a 64-fold reduction in the required COL dose. The consistency of synergy among diverse MDR-GNP suggests that the COL/DRO combination targets a shared bacterial vulnerability, likely linked to membrane integrity. These results establish DRO as a broad-spectrum potentiator of COL activity against clinically significant MDR-GNP. However, when we tested the COL/DRO combination against four clinical colistin-resistant *P. aeruginosa* isolates (COL MIC > 64 µg/mL) (28), no synergistic effect was observed (Table 2), which is consistent with our proposed mechanism of action.

**TABLE 2:** MIC (μg/mL) of dronedarone, colistin, and their combinations against colistin-resistant *P. aeruginosa* isolates

Strain	Colistin		Dronedarone		Interpretation
	Alone	In combination	Alone	In combination	
*P. aeruginosa* 1125	>64	>64	>64	>64	Indifference
*P. aeruginosa* 1131	>64	>64	>64	>64	Indifference
*P. aeruginosa* 1133	>64	>64	>64	>64	Indifference
*P. aeruginosa* 1571	>64	>64	>64	>64	Indifference

### The killing kinetics of COL/DRO combination against carbapenem-resistant MDR-GNP

**Fig 3:**
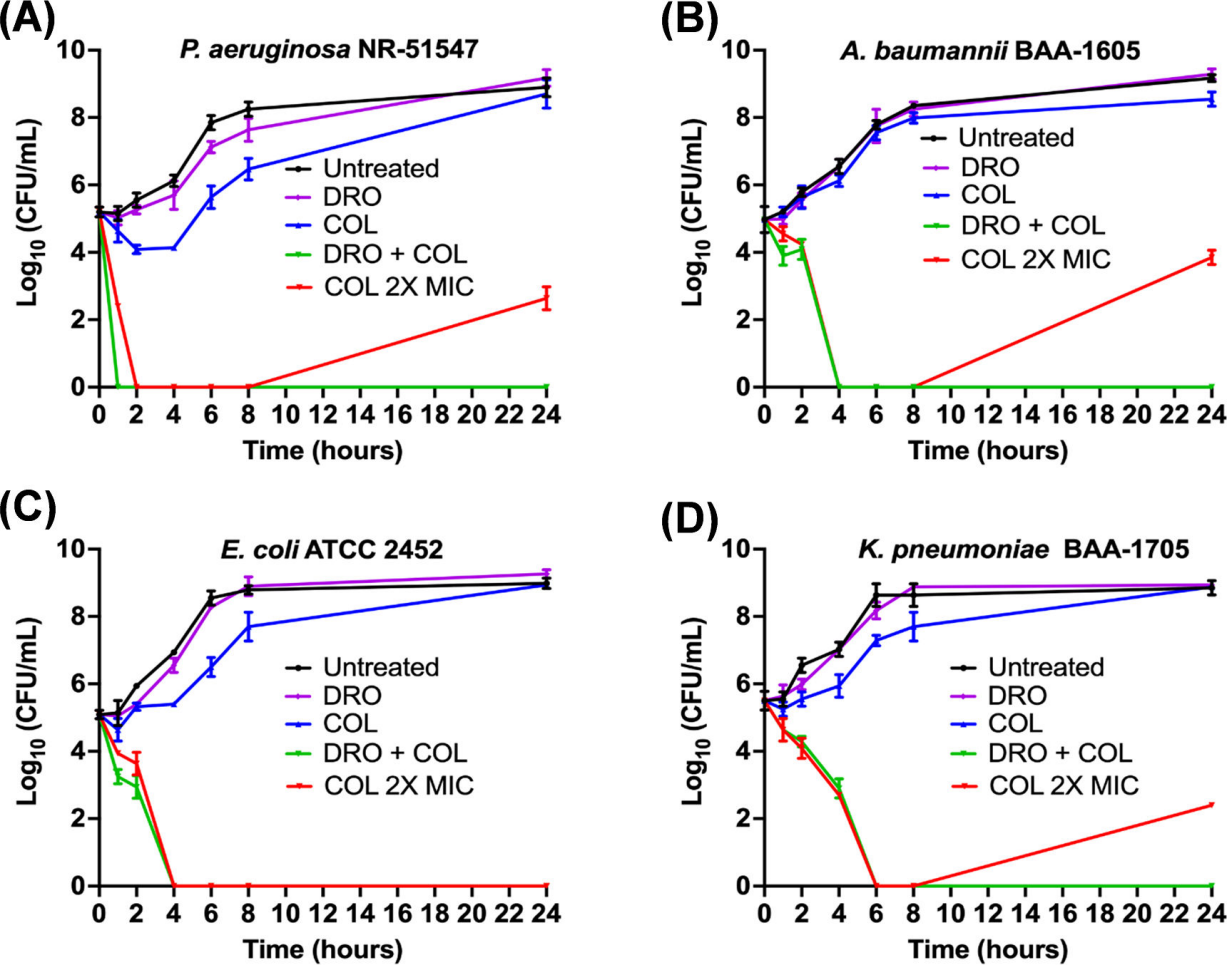
Time-kill assay demonstrating the efficacy of the COL/DRO combination against carbapenem-resistant MDR-GNP. Bacteria were treated with DRO (8 µg/mL) alone and in combination with COL (0.25× MIC). Bacteria were also treated with COL at 2× MIC alone. (**A**) *P. aeruginosa* NR51547, (**B**) *A. baumannii* BAA-1605, (**C**) *E. coli* BAA-2452 and (**D**) *K. pneumoniae* BAA-1705. Experiments were done in three biological replicates per each treatment. Data are shown as the mean ± SEM (*N* = 3 replicates).

To further assess the bactericidal activity of the COL/DRO combination, time-kill kinetics were examined. When tested individually, DRO (8 µg/mL) did not inhibit bacterial growth, and COL at sub-inhibitory concentrations (0.25 × MIC) had no measurable bactericidal effect. However, combining COL and DRO completely restored antibacterial activity, resulting in rapid and complete elimination of all four MDR-GNP within 1–6 h (>6 log reduction) (Fig. 3). The rate of killing varied slightly among species, with *P. aeruginosa* being eradicated within 1 h, *A. baumannii* and *K. pneumoniae* being eradicated within 4 h, while *E. coli* required up to 6 h. Notably, treatment with COL alone at 2× MIC led to significant bacterial killing initially, but regrowth was observed after 24 h in three of the tested isolates (*P. aeruginosa*, *A. baumannii,* and *K. pneumoniae*), suggesting tolerance or adaptive resistance. In contrast, no regrowth was observed in any of the COL/DRO-treated cultures, indicating a durable and sustained bactericidal effect. These findings imply that DRO not only enhances COL activity but may also prevent the emergence of resistant subpopulations during therapy.

### DRO did not increase the cytotoxicity of COL against Vero cells and human RBCs

**Fig 4:**
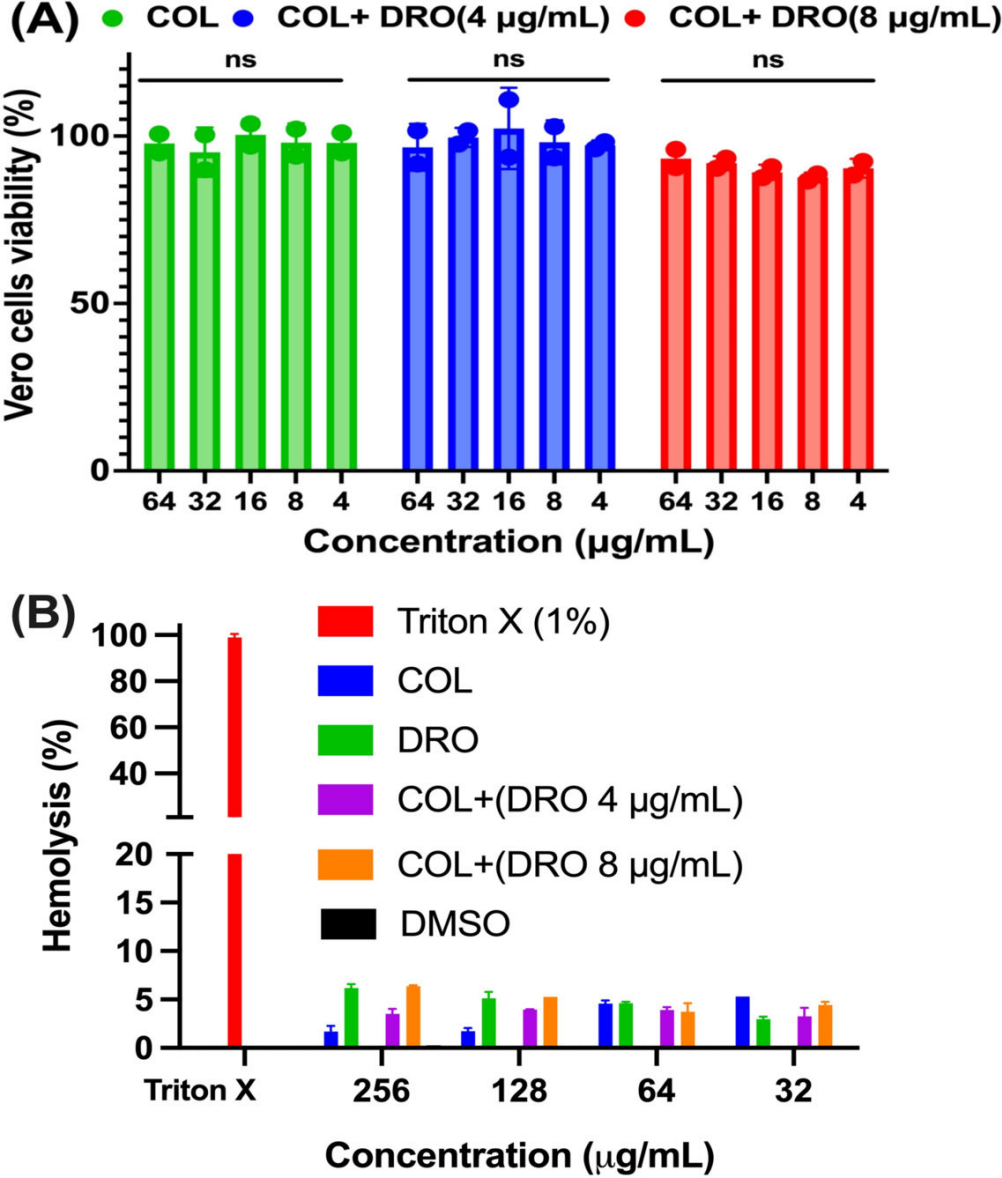
*In vitro* cytotoxicity of COL in presence or absence of DRO against Vero cells (**A**) and human RBCs (**B**). Triton X (1%) caused 100% hemolysis. Statistical analyses were determined by one-way ANOVA with post hoc testing. ns, not significant. Data are shown as the mean ± SEM (*N* = 3 replicates).

The safety profile of the COL/DRO combination was evaluated to ensure selective antibacterial activity. Treatment of Vero cells (monkey kidney epithelial cells) with COL/DRO revealed no significant increase in cytotoxicity compared to COL alone (Fig. 4A). The observed cell viability exceeded 90% across all tested concentrations, confirming that DRO does not enhance COL-associated toxicity. Similarly, hemolysis assays using human red blood cells showed that DRO exhibited negligible hemolytic activity up to 256 μg/mL (<5%), and no enhanced hemolysis was detected in samples treated with the COL/DRO combination (Fig. 4B). These results demonstrate that the synergistic antibacterial effect is specific to bacterial cells and is not due to generalized membrane damage or toxicity, supporting the therapeutic potential of this combination for systemic use.

### The potential mechanism of action/synergy of COL/DRO

**Fig 5:**
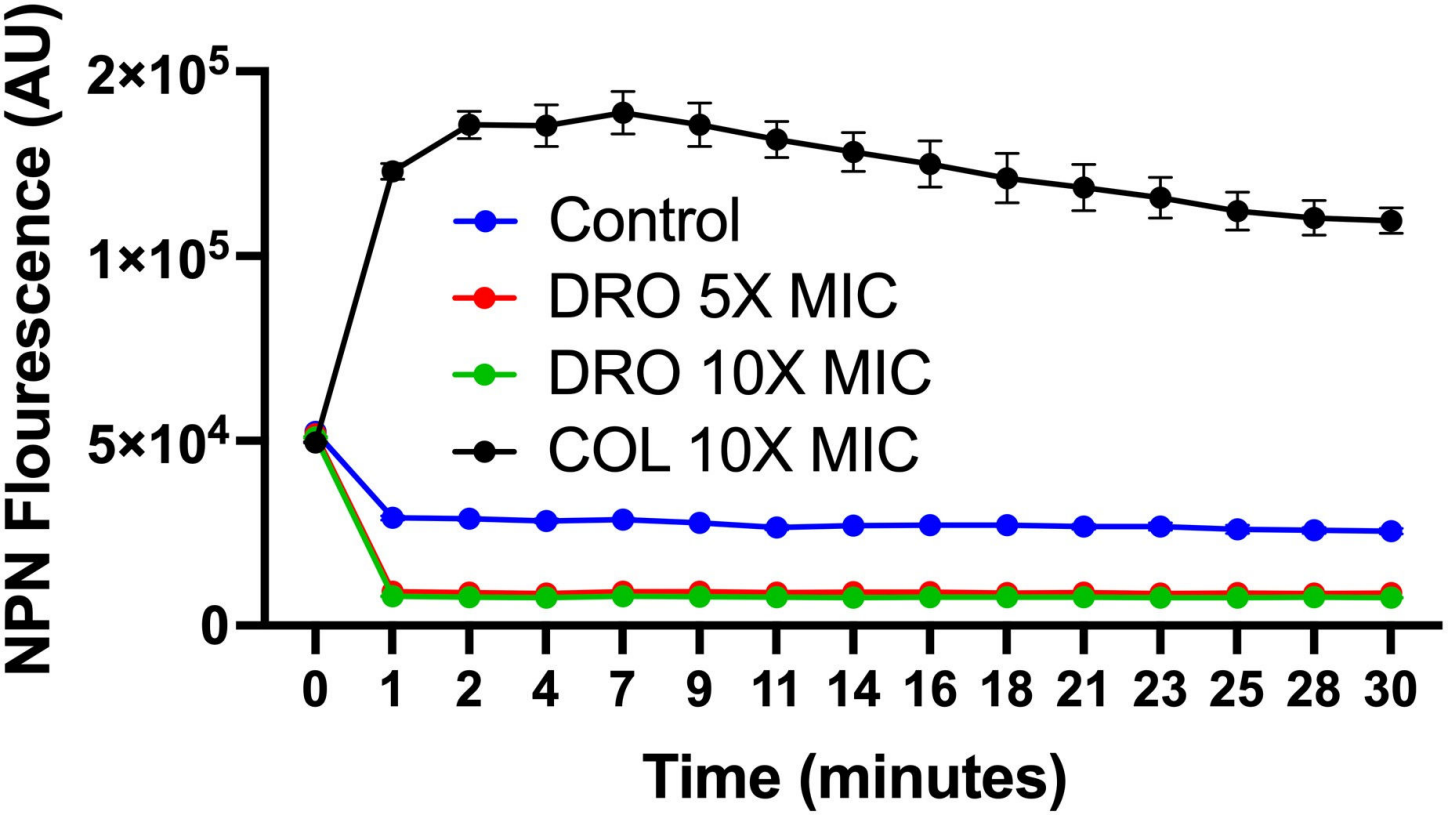
DRO did not induce permeabilization of the outer membrane of *E. coli*, whereas COL did, as evidenced by the increased fluorescence of NPN in the COL-treated sample. Data are shown as the mean ± SEM (*N* = 3 replicates).

Intrigued by the synergy of the COL/DRO combination, we conducted a series of experiments to identify the potential mode of action. We first assessed damage to the bacterial outer membrane (OM) by monitoring the fluorescence intensity in *E. coli* mixed with 1-*N*-phenylnaphthylamine (NPN), a dye that increases fluorescence upon partitioning into the OM (30, 31). Our results (Fig. 5) revealed a significant increase in fluorescence intensity in *E. coli* cultures treated with COL, while DRO alone did not elicit a similar response (similar results were observed with *P. aeruginosa*). Based on these findings, we hypothesized that COL effectively disrupts the OM, thereby facilitating DRO’s access to its bacterial target. To test this hypothesis, we examined the antibacterial activity of DRO against *E. coli* following chemical perturbation of the OM using polymyxin B nonapeptide (35), a less potent analog of polymyxin B that selectively disrupts the OM without antibacterial properties (MIC > 100 μg/mL). While DRO was inactive against WT *E. coli* (>256 µg/mL), it became active (MIC of 4 μg/mL) when combined with polymyxin B nonapeptide. Similar outcomes were observed with *P. aeruginosa* and *A. baumannii* as detailed in Table 3.

**TABLE 3:** Polymyxin B nonapeptide (PMBN) enhances the antimicrobial efficacy of DRO

Drug	MIC (μg/mL)					
	*P. aeruginosa*		*A. baumannii*		*E. coli*	
PMBN	−	+	−	+	−	+
Dronedarone	>256	4	256	4	>256	4
Vancomycin	128	16	128	16	128	16
Amikacin	4	2	4	2	>64	>64
Colistin	0.25	0.125	0.125	0.06	0.125	0.06
PMBN	>50	>50	>50	>50	>50	>50

We further explored if altering the OM through genetic manipulation would yield comparable outcomes by examining how DRO performed against a strain with compromised OM integrity. In a strain of *E. coli* with a ΔbamBΔtolC deletion (29), resulting in high permeability to small molecules due to the absence of BamB (a crucial element of the β-barrel assembly mechanism for OM proteins) and TolC (an efflux channel in the outer membrane), DRO exhibited an MIC of 4 μg/mL (as detailed in Table 4). These results suggest that enhancing OM permeability is sufficient to allow DRO to reach and act on its bacterial target.

**TABLE 4:** MIC (μg/mL) of drugs and control antibiotics against WT *E. coli* and its mutant

Drug	MIC (μg/mL)	
	WT *E. coli*	*E. coli* Δ*bamB*Δ*tolC*
Dronedarone	>256	4
Colistin	0.125	0.06
Vancomycin	128	4
Gentamicin	0.25	0.125

**Fig 6:**
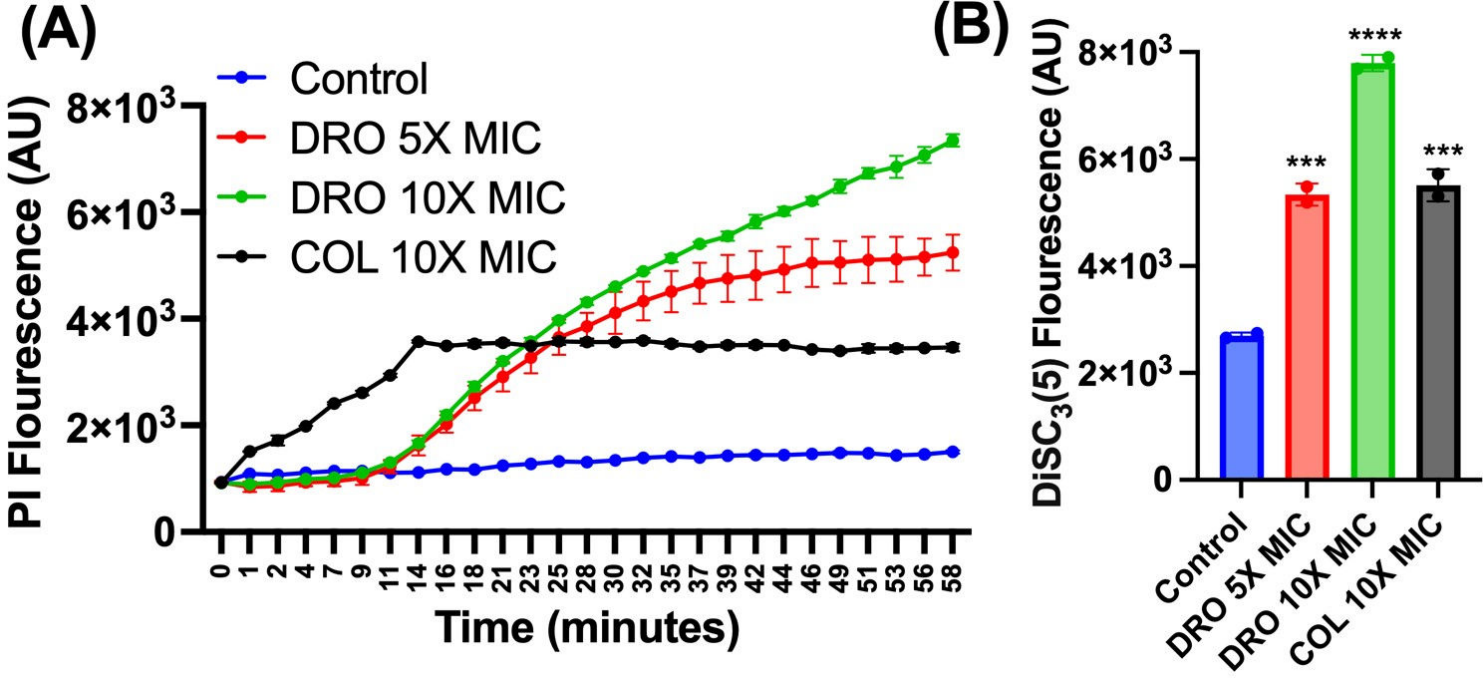
DRO- induced permeabilization (**A**) and depolarization (**B**) of the cytoplasmic membrane of *E. coli* Δ*bamB*Δ*tolC*, as indicated by the elevated fluorescence of PI and DiSC3(5), respectively. Data are shown as the mean ± SEM (*N* = 3 replicates). Statistical analyses were determined by one-way ANOVA with post hoc testing (***P < 0.005), (****P < 0.0001).

Finally, we examined the impact of DRO on the inner membrane function and integrity using *E. coli* strain Δ*bamB*Δ*tolC* (MIC = 4 µg/mL). This was achieved by utilizing the membrane potential-sensitive dye (DiSC3(5)) (36), and the cell-impermeable propidium iodide (PI) (30, 31). Interestingly, DRO caused a dose-dependent increase in fluorescence intensity, indicating that DRO disrupted the membrane potential and compromised the cytoplasmic membrane integrity (Fig. 6). These findings suggest that COL’s disruption of the OM facilitates DRO entry, leading to inner membrane permeabilization and subsequent bacterial death, which likely explains the observed synergy between the two compounds.

### The COL/DRO combination significantly eradicated established biofilm of all four MDR-GNP

**Fig 7:**
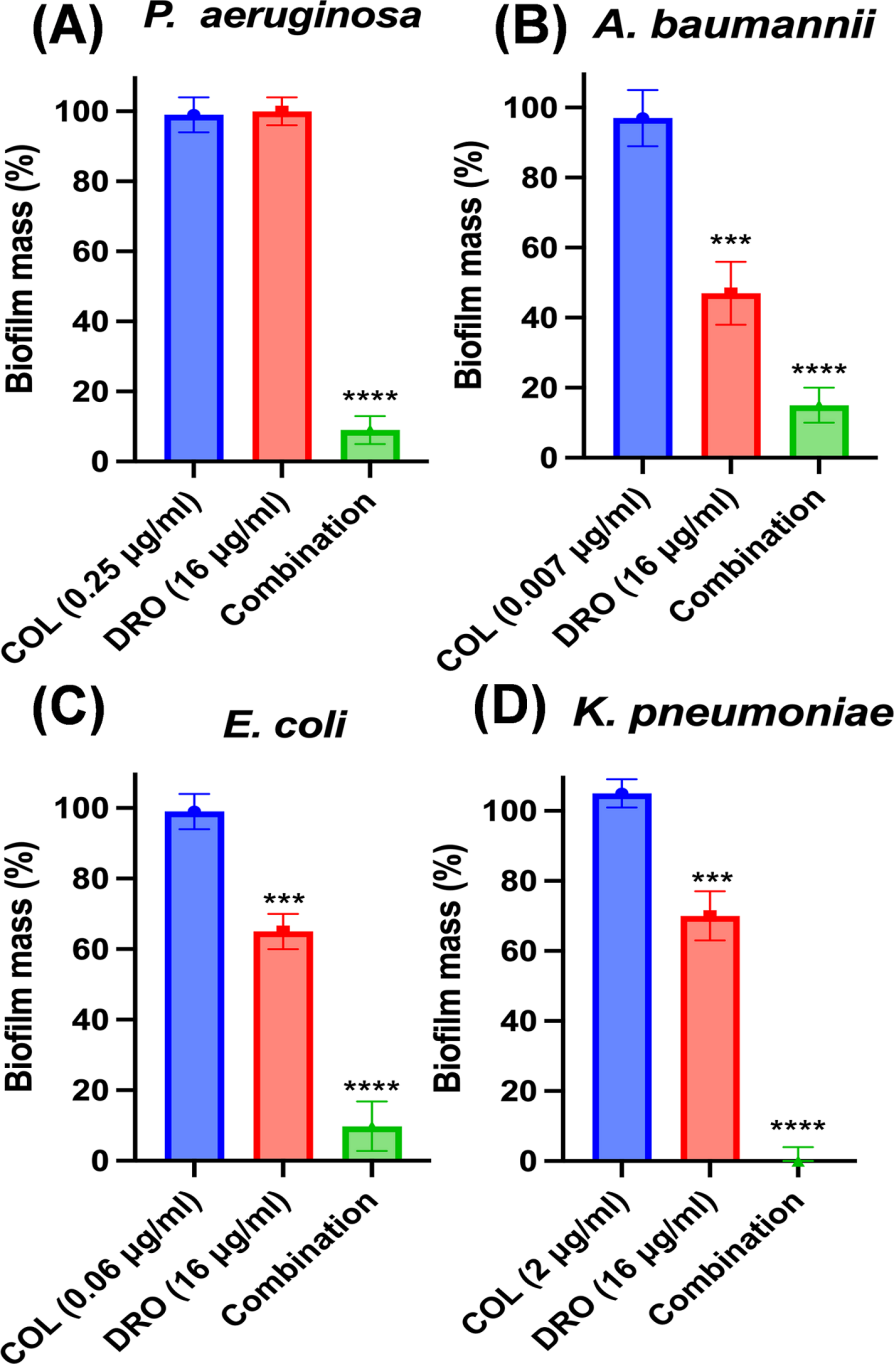
The COL/DRO combination significantly eradicated established biofilm of all four carbapenem-resistant MDR-GNP. (**A**) *P. aeruginosa* NR51547, (**B**) *A. baumannii* BAA-1605, (**C**) *E. coli* BAA-2452 and (**D**) *K. pneumoniae* BAA-1705. Statistical analyses were determined by one-way ANOVA with post hoc testing (***P < 0.005), (****P < 0.0001). Data are shown as the mean ± SEM (*N* = 3 replicates).

Biofilm formation contributes to chronic and recalcitrant infections by MDR-GNP, rendering them highly tolerant to traditional antibiotics and host immune responses (37). Therefore, we evaluated the efficacy of the COL/DRO combination against preformed biofilms. Quantitative biofilm assays revealed that the COL/DRO combination disrupted more than 90% of established biofilms in all tested pathogens (*P. aeruginosa*, *A. baumannii*, *K. pneumoniae*, and *E. coli*) (*P* < 0.0001; Fig. 7 and Table 5). In contrast, COL alone had minimal impact, while DRO alone reduced biofilm biomass by 30%–50% in three of the species tested, *A. baumannii*, *K. pneumoniae*, and *E. coli* (*P < 0.005*) but had limited effect on *P. aeruginosa*. This partial antibiofilm activity of DRO, despite its lack of planktonic antibacterial effect, suggests that DRO interferes with biofilm matrix integrity or bacterial adhesion. The pronounced synergistic antibiofilm effect of the combination underscores its potential in treating infections associated with indwelling devices and persistent biofilm-related infections.

**TABLE 5:** OD_550_ values of biofilm treated samples

	Control	COL	DRO	Combination
*P. aeruginosa*	0.234	0.246	0.231	0.082
	0.24	0.22	0.216	0.078
*A. baumannii*	0.401	0.374	0.227	0.05
	0.369	0.369	0.24	0.07
*E. coli*	0.485	0.476	0.334	0.029
	0.49	0.493	0.376	0.035
*K. pneumoniae*	0.459	0.421	0.34	0.003
	0.472	0.457	0.343	0.001

### *In vivo* efficacy of the COL/DRO combination in a *C. elegans* infection model

**Fig 8:**
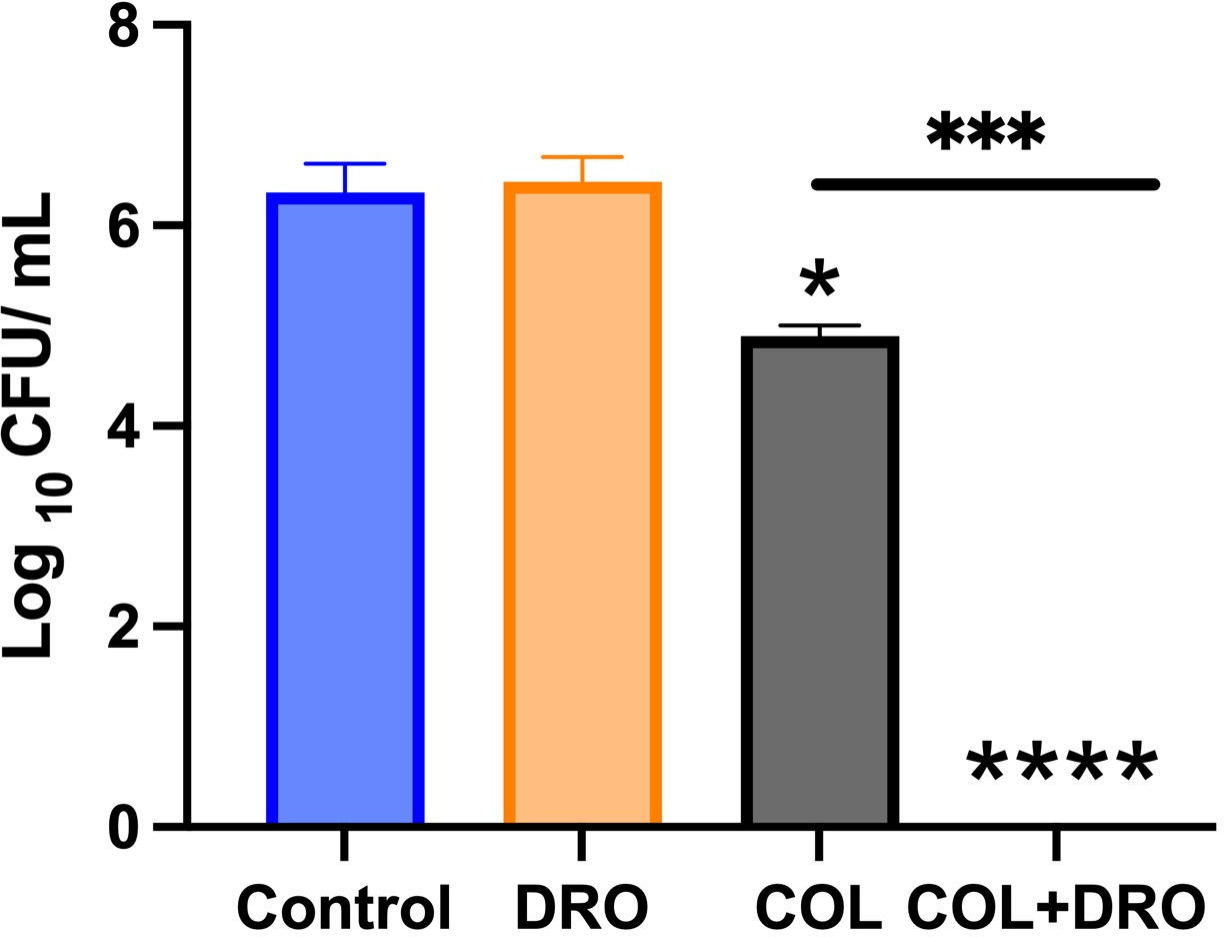
Efficacy of drugs in a *Caenorhabditis elegans* model of bacterial infection. *C. elegans* were infected with carbapenem-resistant *P. aeruginosa* NR 51547. After infection, worms were treated with DRO (12 µg/mL), COL (1 µg/mL), or a combination of both. After 20 h, worms were lysed and bacteria were plated on *Pseudomonas* isolation agar and CFU were counted after 24 h. Results are expressed as means from three biological replicates ± standard deviation. Statistical analyses were determined by one-way ANOVA with post hoc testing (*P < 0.05), (***P < 0.005), (****P < 0.0001).

To evaluate whether the observed *in vitro* synergy translates to an *in vivo* setting, we tested the efficacy of the COL/DRO combination in a *C. elegans* infection model (38–40) infected with carbapenem-resistant *P. aeruginosa* NR51547. Treatment with DRO (12 µg/mL) alone did not significantly affect bacterial burden, whereas COL (1 µg/mL) resulted in a modest 1-log reduction in colony counts (*P < 0.05*). Remarkably, the combination of COL and DRO resulted in complete bacterial eradication (>6 log reduction, *P* < 0.0001) within 24 h (Fig. 8). Importantly, worms treated with the combination remained viable and active throughout the assay, indicating that the treatment was not toxic to the host. These results provide compelling *in vivo* evidence that the COL/DRO combination restores colistin efficacy and effectively clears infections caused by highly resistant *P. aeruginosa*.

## DISCUSSION

The emergence and global spread of MDR-GNP pose an urgent threat to public health, limiting effective treatment options and elevating mortality rates (1–4). COL, a last-resort antibiotic, is often the only therapeutic option available for treating infections caused by these resistant pathogens. However, the clinical utility of COL is severely restricted by its dose-limiting nephrotoxicity and the alarming rise of COL-resistant strains (5, 6). Thus, strategies aimed at enhancing the efficacy of COL while minimizing its toxicity are critically needed. In this study, we report the identification of DRO, an FDA-approved antiarrhythmic agent, as a potent synergistic partner with COL against MDR-GNP, including *P. aeruginosa*, *A. baumannii*, *K. pneumoniae*, and *E. coli*.

Our screening of ~3,400 FDA-approved drugs led to the identification of DRO as a COL potentiator, restoring its activity against *P. aeruginosa*. Notably, DRO alone displayed no intrinsic antibacterial activity, suggesting that its mechanism is entirely dependent on its synergistic interaction with COL. *In vitro* synergy was confirmed by checkerboard assays across several MDR-GNP strains, with fractional inhibitory concentration ranging from 0.021 to 0.13 indicating strong synergy. Time-kill assays further confirmed that while COL or DRO alone were ineffective at subinhibitory concentrations, their combination resulted in rapid and complete bacterial killing, with no observed regrowth at 24 h. This suppression of regrowth may indicate a potential for resistance prevention, an especially valuable trait given the rapid emergence of resistance to many existing antibiotics.

The COL/DRO combination also demonstrated remarkable activity against mature biofilms. Biofilm-associated infections represent a major clinical hurdle due to their increased tolerance to host immune responses and antibiotics (41, 42). While COL alone failed to eradicate established biofilms, the COL/DRO combination disrupted more than 90% of biofilm mass in all tested MDR-GNP. Interestingly, DRO alone exhibited modest antibiofilm activity despite lacking direct antibacterial effects, possibly reflecting interference with biofilm structural integrity or quorum sensing mechanisms. These findings are highly encouraging, as they suggest the potential utility of this combination in treating chronic and device-associated infections where biofilms are prevalent (37).

Mechanistic studies revealed that COL acts by compromising the outer membrane (OM) of Gram-negative bacteria, which, in turn, facilitates the entry of DRO. Once inside the periplasmic space, DRO targets the inner membrane, inducing membrane depolarization and permeability, as evidenced by assays using fluorescent dyes and genetically modified *E. coli* strains with compromised OM. This two-step mechanism—outer membrane disruption by COL followed by inner membrane damage by DRO—underlies the observed synergistic bactericidal effect. Importantly, this dual-targeted approach may reduce the likelihood of resistance development, as bacteria would need to acquire simultaneous protection against both membrane insults (43, 44).

In our study, we evaluated the combination of DRO and COL against clinical colistin-resistant isolates of *P. aeruginosa*. No synergistic interaction was observed, which aligns with the high-level resistance profile of these isolates (MIC > 64 µg/mL). This outcome appears consistent with our proposed mechanism of action, in which colistin disrupts the outer membrane to facilitate dronedarone entry and subsequent disruption of the inner membrane. Interestingly, a recent paper by Liu et al. (45) reported a synergistic effect between DRO and COL against colistin-resistant *E. coli* and *Salmonella* strains. The key distinction between the two studies likely lies in the bacterial species tested and the degree of COL resistance. The *E. coli* isolates used by Liu et al. exhibited moderate resistance (MIC = 4–8 µg/mL) (45), whereas the *P. aeruginosa* isolates in our study displayed much higher resistance levels (MIC > 64 µg/mL). These findings collectively suggest that dronedarone may potentiate colistin activity against strains with low-to-moderate resistance but is less effective against those with high-level resistance. Further investigations involving a broader panel of clinical isolates with varying resistance levels are warranted to confirm this observation and to better define the threshold at which synergism is maintained or lost.

Safety is a major concern in combination therapies, particularly when repurposing non-antibiotic agents (44, 46, 47). In this study, DRO did not increase the cytotoxicity of COL in mammalian cell models, including Vero cells and human red blood cells. This selective toxicity toward bacterial cells supports the translational feasibility of the COL/DRO combination. Furthermore, DRO is already used clinically in humans at relatively high doses for the management of cardiac arrhythmias, with a well-characterized safety and pharmacokinetic profile (13, 14). Its broad tissue distribution, particularly to the lungs and kidneys (14)—organs commonly involved in MDR-GNP infections—makes DRO an attractive candidate for repurposing in infectious disease therapy.

To further support the clinical potential of this combination, we employed an *in vivo C. elegans* infection model. This model demonstrated a profound synergistic effect: while DRO or COL alone had limited efficacy, their combination resulted in complete eradication of carbapenem-resistant *P. aeruginosa* from infected nematodes. These results strongly suggest that the synergy observed *in vitro* translates to biological efficacy in a whole-organism context.

Beyond its potent *in vitro* and *in vivo* antibacterial activity, the COL/DRO combination holds promise in addressing one of the most pressing clinical concerns, limiting the emergence of resistance during therapy. Combination regimens that target distinct bacterial structures or processes, such as the dual membrane-disruption strategy described here, have been shown to reduce the probability of resistance selection compared to monotherapies (43, 44, 47). The lack of regrowth observed in time-kill assays with COL/DRO, in contrast to COL alone, further supports this potential. This feature could be particularly beneficial in treating patients with prolonged infections, recurrent episodes, or in immunocompromised populations where bacterial clearance is challenging (47). Moreover, the inherent antibiofilm activity of DRO could extend the utility of this combination to device-related infections, such as those involving ventilator tubing, urinary catheters, or prosthetic implants—clinical scenarios where biofilm-mediated tolerance often leads to persistent colonization and treatment failure (42, 48, 49).

Future research should explore the translational potential of the COL/DRO combination in murine models of infection, with a focus on pharmacokinetics, tissue penetration, and toxicity at clinically relevant dosing regimens (50). Given DRO’s established human safety profile for cardiovascular indications, dose optimization studies could be accelerated toward clinical application (50). Consistent with our *in vitro* and *in vivo* findings, Liu et al. recently reported that DRO significantly potentiated COL activity in a murine infection model of COL-resistant *E. coli*, thereby underscoring the translational value and supporting the future evaluation of COL/DRO regimen in relevant animal models of other Gram-negative pathogens (45). Additionally, examining the efficacy of this combination against other problematic Gram-negative pathogens, including *Enterobacter* spp. and *Stenotrophomonas maltophilia*, may broaden its therapeutic relevance (51, 52). Investigating potential interactions with standard-of-care antibiotics beyond COL could uncover further synergistic opportunities.

In conclusion, this study highlights the potential of DRO as a novel potentiator of COL in the fight against MDR-GNP infections. The COL/DRO combination shows potent synergy reducing effective drug doses, eradicating biofilms, and exhibiting selective toxicity toward bacteria. Given the growing threat posed by MDR-GNP and the lack of new antibiotic classes in development, this repurposing strategy represents a timely and impactful approach. Further studies are warranted to evaluate the pharmacokinetics, safety, and efficacy of this combination in murine models and eventually in clinical settings.
